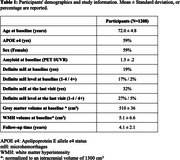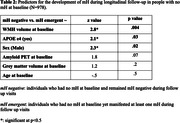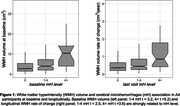# Elevated baseline white matter hyperintensity volume is related to the emergence of microhemorrhages in preclinical Alzheimer’s disease: the A4 Trial

**DOI:** 10.1002/alz.095002

**Published:** 2025-01-09

**Authors:** Zahra Shirzadi, Aaron P Schultz, Adam M. Brickman, Michael Rafii, Steven M. Greenberg, Rema Raman, Paul S. Aisen, Reisa A Sperling, Jasmeer P. Chhatwal, A4 STUDY Team

**Affiliations:** ^1^ Massachusetts General Hospital, Harvard Medical School, Boston, MA USA; ^2^ Taub Institute for Research on Alzheimer’s Disease and the Aging Brain, Columbia University, New York, NY USA; ^3^ Alzheimer’s Therapeutic Research Institute, University of Southern California, San Diego, CA USA

## Abstract

**Background:**

Increased white matter hyperintensity volume (WMH) visible on MRI is a common finding in Alzheimer’s disease (AD) and is often attributed to small vessel ischemic changes. We hypothesized that WMH in preclinical AD is associated with worsening of vessel amyloidosis manifested as microhemorrhages (mH, ARIA‐H). We examine this hypothesis using cross‐sectional and longitudinal data over 4.5 years from the Anti‐Amyloid Treatment in Asymptomatic Alzheimer’s study (A4).

**Method:**

MRI data from N = 1208 older adults were available at baseline (Table 1). MRI data from 1112 participants were available longitudinally (number of sessions = 5945). We extracted WMH using HyperMapper (https://hypermapp3r.readthedocs.io/). Definite mH were identified by experienced radiologists at Mayo Clinic. We categorized mH based on presence (no/yes) and by amount (level: 0, 1‐4, 4+). Linear regression compared WMH at baseline with respect to mH presence and level. We then assessed the relationships between longitudinal WMH accumulation (obtained from a linear mixed effect model) and last visit mH. Lastly, we assessed the relationship between baseline WMH and emergence of mH during follow up using a logistic regression model. All models were corrected for age, sex, grey matter volume, composite cortical amyloid PET, and APOE e4 status.

**Result:**

Baseline WMH volume was greater in individuals with baseline mH compared to those without (t = 4.3, p<0.001). The longitudinal increase in WMH amongst individuals with mH at last visit was estimated to be 141 mm^3^/year greater than those without mH (t = 3.5, p<0.001). Both baseline and longitudinal effects were greater in individuals with more than four mH (Figure 1). Moreover, we observed that higher baseline WMH and APOE e4 status were independently related to emergence of mH during longitudinal follow‐up in A4 participants who did not have mH at baseline (Table 2).

**Conclusion:**

These results suggest a strong link between WMH and ARIA‐H manifested as mH. Notably, baseline WMH was related to future mH emergence in people with no mH at baseline. This suggests increased WMH volume may represent an early sign of vessel amyloidosis, preceding the emergence of mH and thus useful in AD anti‐amyloid treatment planning.